# Towards a common understanding of gender-responsive monitoring and evaluation for health programs and interventions: Evidence from a scoping review

**DOI:** 10.1016/j.ssmhs.2025.100059

**Published:** 2025-03-05

**Authors:** Anna Kalbarczyk, Daniel Krugman, Shatha Elnakib, Elizabeth Hazel, Amy Luo, Anju Malhotra, Rosemary Morgan

**Affiliations:** a Department of International Health, Bloomberg School of Public Health, Baltimore, MD, USA; b Department of Population, Family, and Reproductive Health, Bloomberg School of Public Health, Baltimore, MD, USA; c Global Financing Facility, Washington, DC, USA

**Keywords:** Gender, Monitoring and evaluation, RMNCAHN, Health outcomes

## Abstract

Given the many approaches to and definitions of gender responsive monitoring and evaluation (M&E) for health programs and interventions there is a lack of clarity on how to operationalize it including what to measure and how to measure it. We conducted a scoping review to understand what makes M&E gender responsive. We included 31 studies and conducted two rounds of extraction to delineate ways in which gender was integrated into M&E. Twelve articles described the use of theory to guide M&E though most were not related to gender. Twelve articles employed a gender score in data collection, most of which measured Likert scale responses related to gender equity. Even though most studies did not use a specific gender framework, most incorporated gender domains in their analysis. Seven studies used participatory methods in the design and implementation of M&E. Most studies conducted M&E on programs or interventions that were designed to be gender intentional and related to gender issues. Gender responsive M&E intentionally integrates gender into the M&E process, regardless of how gender-intentional the program or intervention is. Gender dimensions can be identified through gender theories, models, scores, and frameworks to inform tool development, data collection, analysis, and stakeholder engagement processes.

## Introduction

There are different approaches to, and definitions of, gender responsive monitoring and evaluation (M&E) in the context of health programs and interventions. Related terminology used to describe similar concepts includes, for example, gender-intentional, gender-sensitive, and gender-specific M&E. The lack of common understanding is unsurprising given diverse understandings of gender, which itself is multi-faceted, including both gender identity and gender power relations. Gender identity refers to whether a person identifies with being a woman, man, or gender minority individual (Tan et al., 2020). Gender power relations, which can impact gender identity, explore the ways in which gender inequities manifest through interpersonal, communal or societal relations, such as through inequitable: access to resources, roles and practices, norms and beliefs, decision-making power and autonomy, and laws, policies, and institutions (Morgan et al., 2016). Gender is also often incorrectly conflated with biological sex (the biological characteristics such as chromosomes and anatomy that define humans as female, male, or intersex) or equated solely with women and girls (Mauvais-Jarvis et al., 2020).

The complexity of gender is compounded by its intersection with other social stratifiers, such as race and age, its context and time varying nature (i.e., how gender power relations manifest in one context may not be the same as in another context and understanding of gender identify and gender power relations change over time), and its embeddedness within the structures and institutions that encompass health. These definitional complexities lead to complexities in its measurement, including difficulty in teasing out relevant factors that may have an impact on a specific health program or intervention.

M&E is the process of tracking progress and determining effectiveness of a program or intervention. It is important for defining program priorities, resources, and activities and helps inform decision-making and learning, as well as establish accountability. Integrating gender into programs and interventions ensures that the ways in which underlying gender inequities might positively and negatively affect program implementation and outcomes are considered and addressed which helps to ensure program outcomes are achieved, the needs and priorities of specific groups (particularly those that are more marginalized) are met, and programs and interventions do not perpetuate harmful gender inequities.

Operationalizing gender responsive M&E has been variable, and applications often include different components. How gender responsive M&E is operationalized is important, as it affects what actions are taken. For example, within the literature, gender responsive M&E has been described as: 1. measuring underlying gender inequality that leads to poor health outcomes and inequities between and within groups (usually between men and women) (UNAIDS, 2016); 2. whether a program or intervention addresses the different priorities and needs of men and women (EIGE, 2016); 3. whether a program or intervention addresses gender inequities to improve health and wellbeing; and/or 4. measuring and evaluating differential effects of an intervention or program on groups (usually men and women) as well as any unintended consequences that may disadvantage one group over the other (UN Women, 2020). Some definitions include process-related considerations, including how and by whom data is collected and analyzed, and/or whether a M&E process is inclusive and participatory of diverse voices, particularly women’s voices (UN Women, 2020). Many definitions include a combination of the above.

Consequentially, within gender responsive M&E, there are different conceptualizations of what indicators should be used to track progress and determine effectiveness. Indicators range in types, including sex-specific (focusing on women or men or sub-groups of women and men), sex-disaggregated (comparing women or men), or gender equality indicators (unequal access to resource, agency, rights, and power relations)that leads to poor health outcomes and inequities) (UNAIDS, 2016). Some view the inclusion of sex/gender disaggregated data or indicators as sufficient to be considered gender responsive, while others think that gender M&E indicators need to go beyond sex/gender disaggregation to measure gender inequality and progress towards gender equality (UNDP, 2020). The overall lack of consensus on and operationalization of gender responsive M&E indicators has stymied clarity and integration of gender within programs and interventions (Morgan et al., 2022).

Considering these differing approaches, we conducted an exploratory scoping review of the academic peer reviewed literature to examine the nature of how gender has been incorporated into the M&E of health programs and interventions, with a particular focus on reproductive, maternal, newborn, children and adolescent health, and nutrition (RMNCAHN). The review was conducted as part of the Monitoring and Action for Gender and Equity (MAGE) project (a collaboration between the Global Financing Facility and Johns Hopkins University) which aims to galvanize gender responsive M&E for RMNCAHN in the context of cisgender women’s and girl’s health. The aim of this review is therefore to develop a consolidated evidence base of how gender (mapped onto the definition of gender power relations above) has been integrated into M&E for RMNCAHN programs and interventions broadly to better inform gender responsive M&E, with the understanding that different (and often inconsistent) terminology and approaches have been used. Ultimately, we wanted to answer the question: what makes M&E gender responsive within RMNCAHN programs?

## Methods

We followed the protocol defined by the Preferred Reporting Items for Systematic Review and Meta-Analysis extension for Scoping Review (PRISMA-ScR). This review was not registered in PROSPERO because the platform does not accept registrations for scoping review protocols.

We conducted a scoping review of peer reviewed publications in 3 databases: PubMed, CINAHL, and SCOPUS. The search was developed by creating search terms that corresponded to three concepts, “gender”, “monitoring and evaluation”, and “RMNCAHN”. Search terms were developed in consultation with a global health informationist and faculty with expertise in each of these three areas. The search was conducted on May 27, 2022. The final search strategy is provided in Supplemental File 1.

Articles were included if they were in English, published between 2010 and 2022, focused on RMNCAHN topics, described a M&E component, and incorporated gender. Articles were excluded if they did not meet all inclusion criteria. Given the changing nature of how gender has been understood and integrated into M&E we aimed to capture more recent and contemporary approaches by focusing on publications from the previous 12 years only. This also represents the timeframe during which gender responsive terminology began to appear in the international development space. We did not choose a specific geographical or regional focus as we were particularly interested in different ways in which gender-responsive M&E has been conducted across settings to identify broad approaches to gender-responsive M&E. It is important to note, however, that gender responsive approaches to M&E will need to be context specific, and what is implemented in one setting may not be appropriate or work within another setting.

### Screening process

After removing duplications, references were imported into the Covidence reference manager platform. Two independent reviewers (DK, AK) conducted the title and abstract screening and subsequent full-text review; conflicts were resolved by a third reviewer (RM).

### Data extraction and analysis

Given the novelty of this review and the diverse array of articles that were included under the selection criteria, we developed a two-review extraction process. In the primary extraction, one author (DK) used the extraction tool in Covidence to broadly extricate the different ways the included articles incorporated gender into their M&E processes. Through this, we were able to get a sense of the different ways in which gender was integrated into the M&E of RMNCAHN programs and interventions to gain a better understanding of what gender responsiveness in the context of M&E is. Due to the lack of a common approach to gender responsive M&E, however, many authors did not refer to their M&E processes as being gender responsive or explain how gender was integrated into their M&E.

With this perspective, two authors (RM and AK) then conducted a second round of extraction and exclusion based on emerging and relevant themes in an Excel matrix, delineating different ways in which gender was integrated based on our knowledge of gender integration. This was an iterative process of categorizing data, allowing patterns, meanings, and key concepts to be identified across the documents, and then refining and grouping data into thematic constructs. The cumulative results from these two extractions are featured in the results section.

## Results

### Search results

**Fig. 1.**
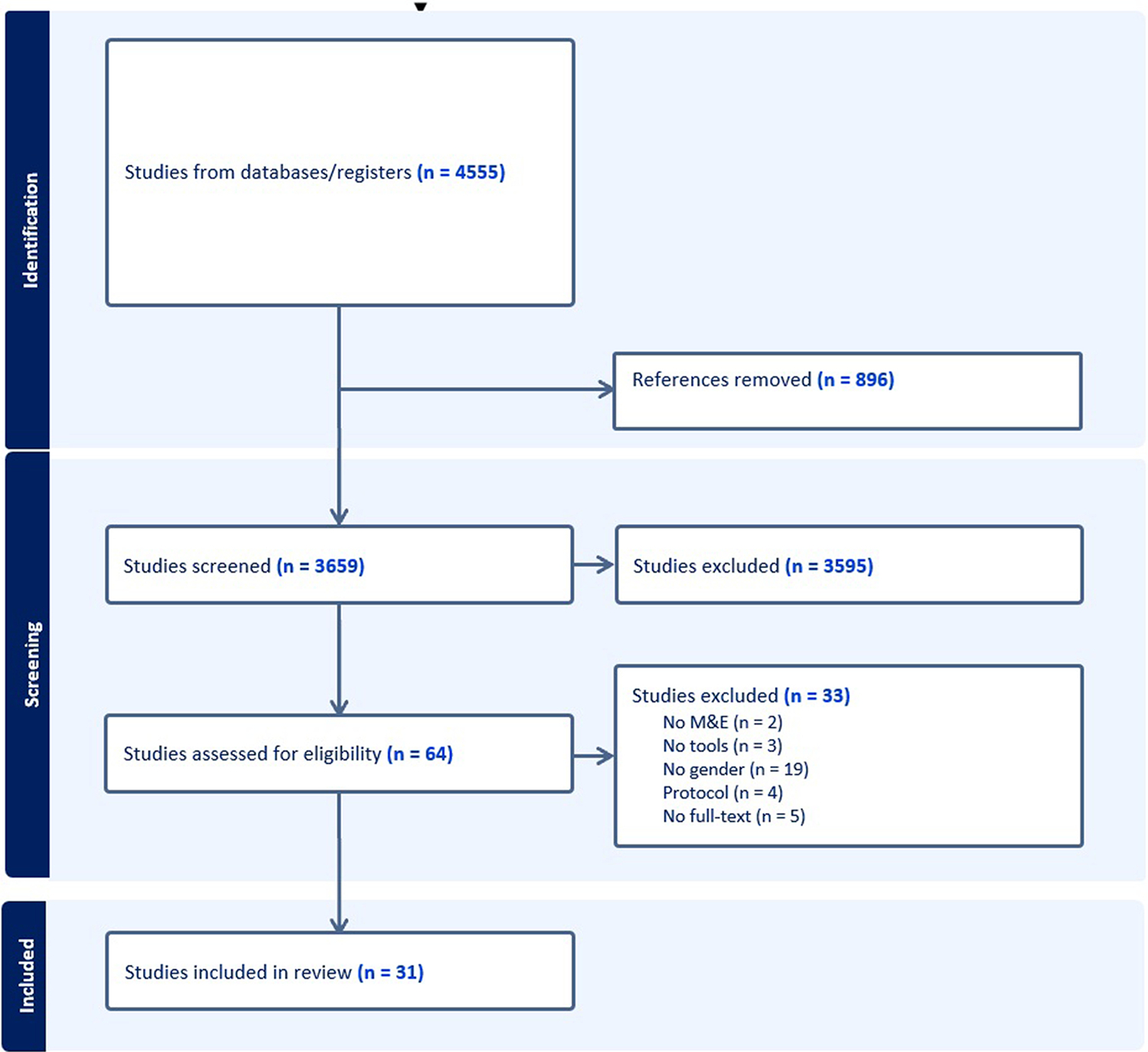
PRISMA Diagram.

We identified 4555 articles which were imported into Endnote for deduplication; 896 duplicates were removed. We screened 3659 references and of those, 3595 references did not meet inclusion criteria. Sixty-four full-texts were then reviewed by two independent reviewers; 33 studies were excluded for not having a gender focus (n = 19), not presenting tools (n = 3), no available full-text (n = 5), protocol only (n = 4), and no M&E (n = 2). Thirty-one articles were included in the final analysis. The PRISMA diagram (Fig. 1) depicts the selection flow diagram.

### Overall description of included studies

Supplemental File 2 outlines the articles included in the extraction. Of the 31 articles that met the inclusion criteria, 26 (84 %) were direct evaluations of interventions (Agam-Bitton et al., 2018; Tura et al., 2020; Speizer et al., 2018; Shimpuku et al., 2019; Seff et al., 2021; Schuster et al., 2019; Santhya et al., 2019; Sahyoun et al., 2019; Muraya et al., 2017; Long et al., 2022; LeCroy et al., 2018; Krishnan et al., 2016; Sharma et al., 2020; Khoza et al., 2018; Burke et al., 2019; Abramsky et al., 2016; Dev et al., 2019; Blanchard et al., 2013; Lees et al., 2021; Exner-Cortens et al., 2021; Figueroa et al., 2016; Berti et al., 2015; Bartels et al., 2019; Bliznashka et al., 2022; Ghanotakis et al., 2017; Colombini et al., 2021), four (13 %) were systematic literature reviews of gender focused methods and evaluation tools (Kowalczyk et al., 2015; Sharma et al., 2022; Adamou et al., 2019; Mandal et al., 2017), and one was a review of a large multi-site program (Pulerwitz et al., 2010). Fourteen of the 26 (45 %) evaluations were conducted in Sub-Saharan Africa (Tura et al., 2020; Speizer et al., 2018; Shimpuku et al., 2019; Seff et al., 2021; Muraya et al., 2017; Khoza et al., 2018; Burke et al., 2019; Abramsky et al., 2016; Dev et al., 2019; Lees et al., 2021; Figueroa et al., 2016; Bliznashka et al., 2022; Ghanotakis et al., 2017; Colombini et al., 2021) while the remaining articles focused on specific locations were split between North America (n = 4) (Long et al., 2022; LeCroy et al., 2018; Exner-Cortens et al., 2021; Berti et al., 2015), the Middle East (n = 4) (Agam-Bitton et al., 2018; Schuster et al., 2019; Sahyoun et al., 2019; Bartels et al., 2019), and India (n = 4) (Santhya et al., 2019; Krishnan et al., 2016; Sharma et al., 2020; Blanchard et al., 2013).

The most prevalent RMANCAH focus area was sexual health (n = 16, 52 %) (Schuster et al., 2019; LeCroy et al., 2018; Krishnan et al., 2016; Sharma et al., 2020; Khoza et al., 2018; Burke et al., 2019; Abramsky et al., 2016; Blanchard et al., 2013; Lees et al., 2021; Exner-Cortens et al., 2021; Figueroa et al., 2016; Bartels et al., 2019; Ghanotakis et al., 2017; Colombini et al., 2021; Mandal et al., 2017; Pulerwitz et al., 2010), largely concerning the issues of gender-based violence (GBV) and intimate partner violence (IPV) (n = 8) (Schuster et al., 2019; Krishnan et al., 2016; Abramsky et al., 2016; Lees et al., 2021; Bartels et al., 2019; Colombini et al., 2021; Sharma et al., 2022; Mandal et al., 2017) and HIV/AIDS prevention (n = 8) (Krishnan et al., 2016; Burke et al., 2019; Blanchard et al., 2013; Figueroa et al., 2016; Ghanotakis et al., 2017; Colombini et al., 2021; Mandal et al., 2017; Pulerwitz et al., 2010). Seven articles focused on maternal and neonatal health interventions (Tura et al., 2020; Shimpuku et al., 2019; Seff et al., 2021; Sahyoun et al., 2019; Muraya et al., 2017; Lees et al., 2021; Berti et al., 2015), ten evaluated adolescent or child health and nutrition programs (Agam-Bitton et al., 2018; Seff et al., 2021; Sahyoun et al., 2019; Muraya et al., 2017; Long et al., 2022; LeCroy et al., 2018; Sharma et al., 2020; Burke et al., 2019; Exner-Cortens et al., 2021; Bliznashka et al., 2022), and three assessed family planning-focused programs (Dev et al., 2019; Ghanotakis et al., 2017; Adamou et al., 2019).

### Types of studies

Most studies employed quantitative methods in the evaluation. Sixteen of 31 studies (52 %) were purely quantitative (Agam-Bitton et al., 2018; Tura et al., 2020; Speizer et al., 2018; Shimpuku et al., 2019; Seff et al., 2021; Schuster et al., 2019; Long et al., 2022; LeCroy et al., 2018; Krishnan et al., 2016; Khoza et al., 2018; Abramsky et al., 2016; Blanchard et al., 2013; Figueroa et al., 2016; Berti et al., 2015; Bliznashka et al., 2022; Ghanotakis et al., 2017), while an additional six studies used qualitative methods as part of a mixed-methods design (Santhya et al., 2019; Sahyoun et al., 2019; Sharma et al., 2020; Burke et al., 2019; Bartels et al., 2019; Pulerwitz et al., 2010). Surveys were the most common instrument used to collect data through randomized control trials (n = 5) (Agam-Bitton et al., 2018; Seff et al., 2021; LeCroy et al., 2018; Khoza et al., 2018; Figueroa et al., 2016), cluster randomized control trials (n = 6) (Tura et al., 2020; Krishnan et al., 2016; Abramsky et al., 2016; Blanchard et al., 2013; Berti et al., 2015; Bliznashka et al., 2022), cross-sectional cohort studies (n = 3) (Shimpuku et al., 2019; Long et al., 2022; Ghanotakis et al., 2017), and controlled cohort studies (n = 1) (Speizer et al., 2018). In the eight mixed-methods and six qualitative-only evaluations (Muraya et al., 2017; Khoza et al., 2018; Dev et al., 2019; Lees et al., 2021; Exner-Cortens et al., 2021; Colombini et al., 2021), the most popular methods employed were in-depth interviewing (n = 7) (Santhya et al., 2019; Muraya et al., 2017; Sharma et al., 2020; Burke et al., 2019; Lees et al., 2021; Exner-Cortens et al., 2021; Colombini et al., 2021), focus group discussions (n = 4) (Muraya et al., 2017; Sharma et al., 2020; Burke et al., 2019; Lees et al., 2021), and semi-structured interviewing (n = 3) (Sahyoun et al., 2019; Dev et al., 2019; Lees et al., 2021). However, two articles employed unique methods surrounding ethnography (Lees et al., 2021; Exner-Cortens et al., 2021). Lees et al. utilized hearsay ethnographies where participants chronicled their daily lives in diaries after receiving training on ethnographic observation (Lees et al., 2021), while Exner-Cortens et al. employed Photovoice, a qualitative research methodology where participants use photography to capture their experiences around a health intervention (Exner-Cortens et al., 2021).

Four articles were systematic reviews of the existing literature (Kowalczyk et al., 2015; Sharma et al., 2022; Adamou et al., 2019; Mandal et al., 2017). Each had different focuses. Adamou et al. explored gaps in male engagement in family planning programs (Adamou et al., 2019). Sharma et al. reviewed “promising practices” for the monitoring of gender-based violence interventions in humanitarian settings around the world (Sharma et al., 2022) More broadly, Mandal et al. reviewed measures of women’s empowerment and other gender-related constructs in family planning and maternal health interventions (Mandal et al., 2017), while Kowalczyk et al. broadly surveyed issues of conducting and evaluating gender-based programs in global health (Kowalczyk et al., 2015). Adamou et al. and Sharma et al. also employed in-depth interviews with their reviews to give more depth to their findings (Sharma et al., 2022; Adamou et al., 2019).

### Ways in which gender was integrated into M&E

Through the data extraction process, we mapped the existing evidence on gender responsiveness in M&E. This included: the use of gender-related theory or frameworks to guide M&E, the inclusion of gender scores, the inclusion of gender domains of analysis, the disaggregation of data by sex, and the use of community-based/participatory approaches.

### Use of gender-related theory, models, or frameworks to guide M&E

Many tools and frameworks used in gender integration are grounded in theory, both gender theory and theory that is relevant for understanding gender. Gender theories include, for example, the theory of gender and power (Gender and Power: Society, the Person, and Sexual Politics – R. W. Connell [Internet], 2023) and male gender norms theory, such as masculinities and hegemonic masculinities (Masculinities by R. W. Connell - Paperback - University of California Press [Internet], 2023). Other cross-cutting theories were used to help contextualize gender as a construct and the ways it manifests, such as intersectionality, the socio-ecological model, life course approach, and social cognitive theory.

Twelve references described the use of some theory or conceptual model to guide the M&E (whether in data collection, development or interpretation of results), though most were not explicitly related to gender. Commonly used models and theories included the Socio-ecological model (SEM) (n = 3) (Seff et al., 2021; Burke et al., 2019; Abramsky et al., 2016) and Social cognitive theory (SCT) (n = 2) (Krishnan et al., 2016; Dev et al., 2019). Kowalczyk et al.’s review on evaluating gender-based programs found that roughly 25 % (n = 32) of the studies included mentioned a conceptual model or theory that guided the research; the most used was SCT (Kowalczyk et al., 2015).

Five studies developed their own guiding frameworks which included a specific gender focus such as male engagement (Adamou et al., 2019), gender norms (Speizer et al., 2018), empowerment (Blanchard et al., 2013), and agency and decision-making (Tura et al., 2020; Muraya et al., 2017). Sharma et al.’s review to identify promising practices for the monitoring and evaluation of GBV interventions highlights the valuable role of a theory of change to guide evaluations, particularly those seeking to assess effectiveness (Sharma et al., 2022).

In Exner-Cortens et al.’s Photovoice study, the authors followed “feminist evaluation principles” to design their community-based participatory research of an empowerment intervention for teenage boys in Canada. They designed the photo-based evaluation method to be participatory, reflexive, and action-oriented to advance feminist goals to move away from patriarchal societal structuring and the perpetuation of misogyny. Aiming to transform the young men into active participants in the effort to reduce gender-based disparities in health through reflecting critically on gender inequity and how to respond to it, they utilized an evaluation that centered gender (Exner-Cortens et al., 2021).

### Incorporation of gender scores into data collection and analysis

Gender scores, or the use of gender variables to quantify the impact of gender as a social construct, measure and elucidate different ways in which gender norms, roles, and relations manifest and associated impact on a particular outcome or outcomes (Nauman et al., 2021). As the way in which gender manifests is multi-faceted and context specific, a standardized set of gender variables is not relevant for all studies. Composite gender scores are sometimes used. As such, many kinds of gender scores exist, with studies often developing their own gender scores relevant for the topic.

Twelve articles employed a gender score tool in their data collection. Often, studies would make use of an established gender score tool, most of which measured Likert scale responses. For example, the Gender Equitable Men (GEM) scale, a theoretically based measure of inequitable and equitable gender norms within sexual and intimate relationships was used in four evaluations (Santhya et al., 2019; Ghanotakis et al., 2017; Adamou et al., 2019; Pulerwitz et al., 2010). This scale was originally developed by Pulerwitz et. al for their study on AIDS interventions in the United States. The GEM scale was also described in Mandal et al. systematic review on measures of women’s empowerment and related gender constructs in family planning and maternal health program evaluations (Mandal et al., 2017). Similarly, Krishnan et al. used the Gender Equity Scale for Women (GESW), an adaption of the GEM, for their study on workplace gender equity in India (Krishnan et al., 2016). Santhya et al. created three additive indices by combining indicators across three domains of interest (index of gender role attitudes and notions of masculinity; index of attendees rejecting men’s controlling behaviors; index of attitudes rejecting violence against women and girls). These indicators were captured using items from the GEM, the WHO multicounty study on violence, the National Family Health survey, and other studies of violence conducted in India (Santhya et al., 2019).

Figueroa et al. developed a gender attitudes scale for their analysis to evaluate an HIV prevention intervention in Mozambique (Figueroa et al., 2016). The scale was based on a series of 12 statements about gender roles assessed using Likert scale responses. Also focusing on gender attitudes, Seff et al. developed a caregiver gender-equitable attitude score to measure the effectiveness of a girl’s education and safety intervention in the Democratic Republic of the Congo (Seff et al., 2021). This score was similarly based on Likert scale responses to 10 statements related to gender roles and dynamics. In their evaluation of a teen sexual confidence intervention Lecroy et al., employed girl efficacy and self-assertive behaviors measures developed in previous studies, also based on sets of Likert items (LeCroy et al., 2018).

Tura et al. used the previously validated Women’s Generalized Self-efficacy scale (GSE) to evaluate agency created by a women’s savings group in Mozambique (Tura et al., 2020). GSE consists of constructive declarations such as “I can always manage to solve difficult problems if I try hard enough”; these are reported on a Likert scale. Continuing the theme of efficacy and agency-focused scores, Sahyoun et al. used both the Women’s Empowerment in Agriculture Index (WEAI) and the Duke Social Support Index (DSSI) (Sahyoun et al., 2019). Evaluating a food and cooking-based employment intervention among refugee women in Palestine, they used the WEAI to assess empowerment of the women and the DSSI to understand the financial and social wellbeing of women through the tool’s open-ended questions. In Mandal et al. Systematic review, authors pinpointed and described the sexual relationships power scale as a key tool to measure determinants or dimensions of women’s empowerment (Mandal et al., 2017).

Finally, Long et al. made use of The Menstruation Engagement, Self-efficacy, and Stress assessment (MENSES), a proven tool that aims to measure girls’ experiences managing menses at school (Long et al., 2022). Used to analyze a menstrual health intervention among young adolescents in Mexico, this tool consists of 45 questions across three outcomes: school-engagement, stress, and self-efficacy.

### Gender domains of analysis

Gender domains of analysis include the different ways in which gender power relations manifest as inequities. A gender analysis framework provides a structure for organizing information about gender power relations. Key domains that constitute these relations include access to resources; division of labor and activities; social norms, ideologies, beliefs, and perceptions; and rules and decision-making (Morgan et al., 2016). These can be incorporated into interview and survey questions, indicators, and variables for analysis. Even though most studies did not use a specific gender framework to guide the M&E, many incorporated gender domains within their analysis, with some using specific tools to assess different gender domains, particularly gender norms.

For example, one study conducted a cultural consensus analysis (CCA) to assess norms among targeted cultural subgroups across the West Bank. Men and women answered questions across the dimensions of GBV, economic empowerment for women, and household and community dynamics of GBV (Schuster et al., 2019). The UZIKWASA project also captured changing norms and practice in relation to key areas of their programs, including early forced marriage, education support, and gender equitable parenting (Lees et al., 2021).

Blanchard et al. used composite empowerment indicators to explore associations between involvement with community mobilization programs, dimensions of self-reported empowerment (defined as power within, power with, and power over), and outcomes of HIV risk reduction (Blanchard et al., 2013). The Sensemaker tool also explored empowerment and decision-making through a complex mixed methods methodology (Bartels et al., 2019). The authors note that this tool, while highly contextualized through co-development, can also be time and labor-intensive in low-literacy environments. It is also one of the few non-Likert scale approaches that were used.

Berti et al. modified a tool from the “Child Survival Technical support project” to assess mothers’ perceptions of father’s role in caring for their pregnant partners and their role in family planning (Berti et al., 2015). Considering gender analysis domains, these measurements can be related to access to resources (i.e. access to male engagement and support), and division of labor.

### Approaches which support gender responsive M&E

Other approaches which support the integration of gender into M&E include the use of sex disaggregated data and community-based/participatory methods. On their own these approaches would not be considered gender responsive, however, they are reported here due to their important role in gender responsive M&E. M&E that only employs disaggregated data by sex or community-based/participatory methods without other dimensions of gender integration would have been excluded in search results.

### Sex disaggregation of data

Sex disaggregation is important to determine whether outcomes differ for different gendered groups. There are three important points of note regarding sex disaggregation. First, a precursor to sex disaggregation is the presence of people of different sexes or genders within the intervention and/or M&E process itself. Second, not all programs or interventions need to include sex disaggregated if they are focused on one sex or gender only (however there may still be need for engagement of other genders). And third, gender differences can be missed if gender domains of analysis, some of which that do not pertain to all genders, are not also included, irrespective of whether sex disaggregated data is used.

Six studies that included both men and women reported sex-disaggregated data (Agam-Bitton et al., 2018; Speizer et al., 2018; Seff et al., 2021; Schuster et al., 2019; Khoza et al., 2018; Abramsky et al., 2016). The MENSES study collected some data from both boys and girls but did not present disaggregated data in their tables (Long et al., 2022). Alternatively, they used socio-economic status to disaggregate data among female respondents. The review conducted by Sharma et al. describes the importance of including participants of various genders and ages as well as disaggregating data based on age, sex, and disability status, to ensure GBV risk mitigation M&E effectively captures potential risks for vulnerable groups. They further add that data analysis should include triangulation of data from multiple sources to assess convergence and divergence of findings (Sharma et al., 2022).

### Meaningful engagement and participation in gender responsive M&E

Some definitions of gender responsive M&E include process-related considerations, such as how data is collected and analyzed, by whom, and/or whether a M&E process is inclusive and participatory of diverse voices, particularly women’s voices. Often such approaches are not described as being gendered unless in reference to feminist methodologies. Such approaches are important because they emphasize collaboration and partnership between investigators and participants, center lived experience as an important form of knowledge, and seek to empower, foster agency, and amplify the voices of participants. This includes considerations for who is engaged and how they are engaged. For example, are gender norms, hierarchies, and power differentials accounted for when designing meetings and other activities? When applied in concert with other gender responsive actions, these approaches can inform how gender and gender-related elements can be integrated within M&E.

Seven studies described the use of community-based or participatory methods in the evaluation design and implementation (Tura et al., 2020; Seff et al., 2021; Schuster et al., 2019; Muraya et al., 2017; Sharma et al., 2020; Lees et al., 2021; Bartels et al., 2019). Five of these studies collaborated with a local non-governmental or civil society organizations in study design (Tura et al., 2020; Bartels et al., 2019) or data collection (Seff et al., 2021; Schuster et al., 2019; Sharma et al., 2020). Two studies worked directly with community members to collect data and interpret data (Muraya et al., 2017; Lees et al., 2021). In Lees’ et al., a male and a female community member trained as primary data collectors gave contextual information for the study. Further, because the study was iterative and dynamic, preliminary findings were discussed with participants in the early stages of the project (Lees et al., 2021).

The systematic review by Sharma et al., found that “building partnerships with key actors and stakeholders, including local groups and organizations” was important to ensuring effective M&E design and implementation (Sharma et al., 2022). The authors further argue that diversity factors must be considered, and M&E should be developed with relevant subgroups in mind, and with an intersectional approach that recognizes gender domains such as power and agency. Promising practices in community engagement included providing mechanisms for community feedback that are not unidirectional and facilitating community dialogues to obtain buy-in.

Sharma et al. also identified positionality of data collectors as an important domain (Sharma et al., 2022). Three studies described positionality considerations in the implementation and write-up of M&E. One manuscript described the positionality of the data collectors, including members of the authorship team (Exner-Cortens et al., 2021). Burke et al. noted that to avoid potential harms, programs need to take steps to ensure that staff do not introduce their own biases, particularly those that reinforce inequitable gender norms (Burke et al., 2019). Seff et al. discussed intentionally using a separate tool for collecting sensitive information from girls, such as those on sexual behaviors and exposure to violence (Seff et al., 2021).

## Discussion

This review identified ways in which gender was integrated into M&E for RMNCAHN programs and interventions. By identifying programs and interventions which intentionally integrated gender into their M&E we were able to elicit specific ways in which gender was considered. These include using relevant theory, models, and frameworks to guide M&E, incorporating gender scores into data collection and analysis, and including gender domains of analysis (which were sometimes guided by frameworks). Additional approaches which support gender responsive M&E include disaggregating data by sex and using community-based and participatory methods, which are both good steps to inform further gender integration but need to be implemented alongside other gender integration activities. We note that there may be other ways in which gender can be integrated into M&E which are not captured here but represent other people’s interpretations of gender responsiveness. We also recognize that there may be M&E that incorporated some of the approaches above but would not have been included within our review if specific gendered language was not used in their descriptions. Our aim was to identify ways in which gender was intentionally considered within M&E to work towards a common understanding of what gender responsive M&E is and how it can be operationalized.

Many M&E approaches included in the review were conducted on programs and interventions which intentionally integrated gender into their design and implementation. Many of the programs and interventions focused on topics such as gender-based violence, sexual health, family planning, and maternal health, and focused on the health and wellbeing of women and girls. Just because a program or intervention focuses on women or girls, however, does not make it gender responsive; this also applies to gender-based violence programs and interventions. Such topics, however, lend themselves well to gender integration, particularly those which are important for ensuring women and girls’ wellbeing, autonomy, and safety. Access to family planning, for example, is vital to ensure women’s reproductive choice, which in turn supports equal opportunity (Mpoyi, 2021). Gender-based violence programs and interventions are important to ensure women’s and sexual and gender minorities’ safety and security, which also help to support equal opportunity (Noble et al., 2017). Given the nature of many of the articles which were included in the review, it became clear that programs which were more gender intentional in their design, more clearly conducted gender responsive M&E. However, gender integrated programs are not necessarily a precursor to gender responsive M&E.

Sex disaggregation is often described as a minimum requirement to conduct gender analysis, however, many RMNCAHN programs and interventions focus exclusively on women and girls, which means that sex disaggregated data may not be applicable. Programs or interventions which focus on one gender are sex specific, which as stated above, are not in and of themselves gender responsive. To include sex disaggregation (to be seen as gender responsive), RMNCAHN programs and interventions sometimes deliberately include men as key decision-makers or heads of households. Male engagement is an important component of gender integration when done correctly in a way that does not perpetuate harmful gender norms (Ruane-McAteer et al., 2020). However, it can become problematic when men’s roles as decision-makers and gatekeepers are reinforced at the expense of women’s autonomy and choice. Regarding M&E, Sharma et al. give the example of interviewing heads of households, which can be problematic when only men are consulted (Sharma et al., 2022).

For programs that are not gender specific, gender disaggregated data is a minimum requirement but not sufficient. Likewise, within gender specific programs, data should be disaggregated by relevant social stratifiers, such as age, race, marital status, etc. (Morgan et al., 2016), however this is also not sufficient for gender integration. It is also not enough that disaggregated data is collected within the M&E process; it also needs to be analyzed in a disaggregated manner and fed into program adaptations and changes where appropriate. Within M&E, disaggregated data is often presented in tables with little to no discussion of the implications for programming, which negates having collected disaggregated data in the first place. Within gender responsive M&E, it is also important to recognize that gender norms change over time, and as such gender integration needs to be able to respond to shifting gender norms to produce meaningful results and ensure sustainability (Heymann et al., 2019). Conducting gender situational analyses, which are an assessment of the gender context and its implications for programming, will help to ensure that gender integration is relevant and context specific.

Gender responsive M&E intentionally integrates gender dimensions into the M&E process. Gender dimensions can be identified through the use of gender theories, models, scores, and frameworks. A program or intervention which has itself integrated gender into its design and implementation will facilitate gender responsive M&E. From our findings, it was clear that programs that were more gender intentional in their design, more clearly conducted gender responsive M&E. Where possible, programs should be designed to integrate gender, however, other ways that gender can be integrated include: (1) using gender theories, models, scores, and/or frameworks to guide tool development; (2) integrating gender domains (which can be guided by gender frameworks) into data collection and analysis; and (3) ensuring meaningful stakeholder engagement throughout the process, which includes gender intentionality in regards to who is engaged and how.

Our review is not without limitations. To be included in our search, references needed to include “gendered” terminology. It is likely that evaluations exist which could be considered gender responsive but do not use gendered terminology to describe their processes and outcomes. This also meant that many of the programs and interventions included in the review themselves integrated gender. We did not include gender minorities in our search terms, limiting potentially relevant studies focusing on gender responsiveness from this lens. Further, our search focused on M&E related to RMNCAHN only but there may be unique findings for M&E of health programs and interventions beyond RMNCAHN. Our search only included peer-review literature and did not capture grey literature and there may be other interesting ways in which gender has been integrated into M&E that has not been published in a peer review journal. We only included literature published in English and may have missed resources published in other languages.

## Conclusion

While gender intentional programs and interventions may facilitate gender responsive M&E, they are neither required nor sufficient. Gender responsive M&E is intentional in its conceptualization, participatory design, tool selection, data collection, analytic approaches, and reporting and use. These findings will be used by the MAGE team to generate guidance on how to enhance M&E through gender responsive processes.

## Supplementary Material

Supplemental Material File 1

Supplemental Material File 2

## Appendix A. Supporting information

Supplementary data associated with this article can be found in the online version at doi:10.1016/j.ssmhs.2025.100059.
