# Supplementary material for: Towards a common understanding of gender-responsive monitoring and evaluation for health programs and interventions: Evidence from a scoping review
Source: SSM Health Syst. Author manuscript; Available in PMC 2026 Feb 27. (PMC12945375; doi:10.1016/j.ssmhs.2025.100059)
Supplement: Supplemental Material File 1 [file NIHMS2149056-supplement-Supplemental_Material_File_1.docx]

**Supplemental File 1: PubMed Search Strategy**

“sexism”[Mesh] OR “gender identity”[Mesh] OR "Personal Autonomy"[Mesh] OR "Relational Autonomy"[Mesh] OR Gender[ti] OR "sex disaggregated"[tiab] OR “gender disaggregated”[tiab] OR "sex differences"[tiab] OR "gender differences"[tiab] OR "women’s participation"[tiab] OR "male engagement"[tiab] OR "couple decision making"[tiab] OR "equitable decision making"[tiab] OR "joint decision making"[tiab] OR "couple communication"[tiab] OR "couple decision-making"[tiab] OR "equitable decision-making"[tiab] OR "joint decision-making"[tiab] OR "sexual relationship power"[tiab] OR "reproductive control"[tiab] OR self-efficacy[tiab] OR "reproductive choice"[tiab] OR "relational autonom*"[tiab] OR "personal autonomy"[tiab] OR "reproductive coercion"[tiab] OR "respectful care"[tiab] OR "respectful maternity care”[tiab] OR "reproductive agency"[tiab] OR “decision-making”[tiab] OR “decision making"[tiab] OR "decision making power"[tiab] OR "decision-making power"[tiab] OR literacy[tiab] OR intersectionality[tiab] OR  "gender roles"[tiab] OR "gender relations"[tiab] OR "gender norms"[tiab] OR "gender power relations"[tiab] OR "gender analysis"[tiab] OR "gender inequit*"[tiab] OR "gender inequal*"[tiab] OR "gender equit*"[tiab] OR "gender equit*"[tiab] OR "gender bias"[tiab] OR "gender gap"[tiab] OR "gender discrimination"[tiab]

AND

“Program Evaluation”[Mesh] OR “Benchmarking”[Mesh] OR “Outcomes and Process Assessment, Health Care”[Mesh] OR “program evaluation”[tiab] OR “program monitoring”[tiab] OR “process monitoring”[tiab] OR “process evaluation”[tiab] OR “outcome and process assessment”[tiab] OR “health monitoring”[tiab] OR “quality assurance, health care”[tiab] OR “health care evaluation mechanisms”[tiab] OR “health information system*”[tiab] OR “HMIS”[tiab] OR “monitoring and evaluation”[tiab] OR "M&E” [tiab] OR “monitoring and evaluation” [tiab]

AND

“Reproductive health”[MESH] OR “reproductive health service”[MESH] OR “family planning”[MESH] OR “family planning services”[MESH] OR Infertility[MESH] OR "Maternal Health"[Mesh] OR "Maternal Health Services"[Mesh] OR “Infant health”[Mesh] OR "Child Health"[Mesh] OR "Child Health Services"[Mesh] OR contraception[MESH] OR “reproductive health”[tiab] OR “contracept*”[tiab] OR “abortion*”[tiab] OR “women’s health”[tiab] OR “sexually transmitted infection*”[tiab] OR “child marriage”[tiab] OR “femicide”[tiab] OR “filicide”[tiab] OR “reproductive rights”[tiab] OR “reproductive health service*”[tiab] OR “Female Circumcision*”[tiab] OR Infibulation*[tiab] OR Clitoridectom*[tiab] OR Clitorectom*[tiab] OR “Female Genital Cutting”[tiab] OR “Female Genital Mutilation*”[tiab] OR “genital cutting”[tiab] OR “Sexual health”[tiab] OR “Sexually transmitted disease*”[tiab] OR “STD*”[tiab] OR “sexually transmitted infection*”[tiab] OR “STI*”[tiab] OR “Human immunodeficiency virus”[tiab] OR “HIV”[tiab] OR “acquired immunodeficiency syndrome”[tiab] OR “acquired immune deficiency syndrome”[tiab] OR “AIDS”[tiab] OR “Vertical transmission”[tiab] OR “mother-to-child transmission”[tiab] OR “mother to child transmission”[tiab] OR “MTCT”[tiab] OR “mother-to-baby transmission”[tiab] OR “mother to baby transmission”[tiab] OR “prevention of mother-to-child transmission”[tiab] OR “prevention of mother to child transmission”[tiab] OR “PMTCT”[tiab] OR “STI testing”[tiab] OR “testing for sexually transmitted infections”[tiab] OR “STD testing”[tiab] OR “testing for sexually transmitted diseases”[tiab] OR “VC”[tiab] OR “voluntary counselling”[tiab] OR “confidential testing”[tiab] OR “STI treatment”[tiab] OR “STD treatment”[tiab] OR “treating STIs”[tiab] OR “treating STDs”[tiab] OR “treating sexually transmitted infection*”[tiab] OR “treating sexually transmitted disease*”[tiab] OR antiretroviral*[tiab] OR “ART”[tiab] OR “antiretroviral therapy”[tiab] OR Menstrua*[tiab] OR “menstrual health”[tiab] OR “menstrual period”[tiab] OR “menstrual education”[tiab] OR “period education”[tiab] OR “menstrual hygiene”[tiab] OR “menstrual cup”[tiab] OR “menstrual flow”[tiab] OR “menstrual suppression”[tiab] OR “menstrual regulation”[tiab] OR “sanitary pad”[tiab] OR tampon[tiab] OR menses[tiab] OR menarche[tiab] OR “family planning”[tiab] OR “reproductive plan*”[tiab] OR contracepti*[tiab] OR “birth control”[tiab] OR “birth spacing”[tiab] OR “child spacing”[tiab] OR condom*[tiab] OR “the pill”[tiab] OR “oral contracepti*”[tiab] OR microbicide[tiab] OR diaphragm[tiab] OR “IUD”[tiab] OR “intrauterine device”[tiab] OR “contraceptive implant”[tiab] OR “progestogen only contraceptive”[tiab] OR “lactational amenorrhea”[tiab] OR “LAM”[tiab] or “postpartum amenorrhea”[tiab] OR “post-partum amenorrhea”[tiab] OR “period abstinence”[tiab] OR “rhythm method”[tiab] OR “calendar method”[tiab] OR “sexual abstinence”[tiab] OR “population control”[tiab] OR “pregnancy prevent*”[tiab] OR “fertility control”[tiab] OR “morning after pill”[tiab] OR “ECP”[tiab] OR “advance* provision”[tiab] OR “self administr*”[tiab] OR infertil*[tiab] OR subfertil*[tiab] OR “assisted reproducti*”[tiab] OR “ART”[tiab] OR “IVF”[tiab] OR “in vitro fertilization”[tiab] OR “in-vitro fertilization”[tiab] OR abortion[tiab] OR “termination of pregnanc*”[tiab] OR “pregnancy termination*”[tiab] OR “vacuum aspiration”[tiab] OR cutterage[tiab] OR “pregnancy loss”[tiab] OR “uterine infection”[tiab] OR “uterine perforation”[tiab] OR “abortion-related complications”[tiab] OR “post-abortion care”[tiab] OR “maternal health”[tiab] OR “Maternal-child health“[tiab] OR “Perinatal Care”[tiab] OR “peri-natal care”[tiab] OR “Postnatal Care”[tiab] OR “post natal care”[tiab] OR “Preconception Care”[tiab] OR  “Prenatal Care”[tiab] OR “pre-natal care”[tiab] OR Pregnan*[tiab] OR “maternal welfare”[tiab] OR obstetric*[tiab] OR “safe motherhood”[tiab] OR “EmOC”[tiab] OR fetus[tiab] OR “IUP”[tiab] OR “unborn child”[tiab] OR prenatal*[tiab] OR antenatal*[tiab] OR“fetal ultrasound”[tiab] OR miscarriage[tiab] OR “fetal loss”[tiab] OR “fetal death”[tiab] OR “fetal therapies”[tiab] OR “fetal monitoring”[tiab] OR birth[tiab] OR childbirth[tiab] OR intrapartum[tiab] OR parturition[tiab] OR perinatal*[tiab] OR labor[tiab] OR labour[tiab] OR delivery[tiab] OR “skilled birth attend*”[tiab] OR “stillbirth”[tiab] OR “caesarian section”[tiab] OR “C-section”[tiab] OR “infant health”[tiab] OR “newborn health”[tiab] OR “new born health”[tiab] OR “baby health”[tiab] OR “babies health”[tiab] OR “postpartum”[tiab] OR “postpartum period”[tiab] OR “postbirth”[tiab] OR postnatal*[tiab] OR “postnatal care”[tiab] OR “postnatal health”[tiab] OR “postnatal service*”[tiab] OR “newborn care”[tiab] OR “neonatal health”[tiab] OR “infant welfare”[tiab] OR “baby health”[tiab] OR “puerperium”[tiab] OR “postpartum contraception”[tiab] OR "child health"[tiab] OR “adolescent sexual health”[tiab] OR “adolescent reproductive health”[tiab] OR “youth sexual health”[tiab] OR “adolescent reproductive health”[tiab] OR “youth reproductive health”[tiab] OR “adolescent health”[tiab] OR “youth health”[tiab] OR “adolescent health services”[tiab] OR “youth friendly services”[tiab] OR “adolescent friendly services”[tiab] OR “pregnant adolescents”[tiab] OR “teenage* pregnanc*”[tiab] OR “Nutrition*”[tiab] OR “food”[tiab] OR “anthropomet*”[tiab] OR “micronutrient*”[tiab] OR “malnutrition”[tiab] OR “malnourished”[tiab] OR “wasting”[tiab] OR “wasted”[tiab] OR “stunting”[tiab] OR “stunted”[tiab] OR “underweight”[tiab] OR “growth”[tiab] OR “breastfeeding”[tiab] OR "complementary feeding"[tiab] OR "dietary diversity"[tiab] OR "meal frequency"[tiab] OR "infant and young child feeding"[tiab] OR “complementary feeding”[tiab] OR “breast feed”[tiab] OR “breastfeeding”[tiab] OR “breast fed”[tiab] OR “complementary food*”[tiab] OR “infant feeding”[tiab] OR “infant and young child feeding”[tiab] OR “maternal nutrition”[tiab] OR “nutrition during pregnancy”[tiab] OR “nutrition in pregnancy”[tiab] OR “child feeding”[tiab] OR “child nutrition”[tiab] OR “infant nutrition”[tiab] OR “micronutrient supplement”[tiab] OR “micronutrient supplements”[tiab] OR “micronutrient supplementation”[tiab] OR “nutrient supplement”[tiab] OR “nutrient supplements”[tiab] OR “nutrient supplementation”[tiab] OR “infant feeding”[tiab] OR “diet”[tiab] OR “dietary intake”[tiab] OR “dietary diversity”[tiab] OR “food intake”[tiab] OR “nutrients”[tiab] OR “vitamins”[tiab] OR “minerals”[tiab] OR “micronutrients”[tiab] OR “macronutrients”[tiab] OR “protein”[tiab] OR “energy”[tiab] OR “undernutrition”[tiab] OR “nutritional deficiency”[tiab] OR “deficiency disorder*”[tiab] OR “overweight”[tiab] OR “obesity”[tiab] OR “weight gain”[tiab] OR “linear growth”[tiab] OR “growth faltering”[tiab] OR “fetal growth restriction”[tiab] OR GBV[tiab] OR "gender based violence"[tiab] OR "intimate partner violence"[tiab] OR "intimate partner abuse"[tiab] OR “domestic violence” [tiab] OR "family violence"[tiab] OR "battered woman"[tiab] OR "spous* abuse"[tiab] OR "partner violence"[tiab] OR "intimate partner abuse"[tiab] OR "sexual violence"[tiab] OR "sexual abuse"[tiab] OR "child marriage"[tiab] OR "forced marriage"[tiab] OR "marital rape"[tiab] OR "violence against women"[tiab] OR “dating violence”[tiab] OR “family violence”[tiab] OR rape[tiab] OR “partner abuse”[tiab] OR “intimate partner abuse”[tiab] OR “domestic abuse”[tiab] OR “wife abuse”[tiab] OR “sex crime”[tiab] OR “sexual crime”[tiab] OR “sexual assault”[tiab] OR “sexual harassment”[tiab] OR “sexual coercion”[tiab] OR “forced sex”[tiab] OR “sexual slavery”[tiab] OR “abused woman”[tiab] OR “abused women”[tiab] OR “battered wom*n”[tiab] OR "Spouse Abuse"[Mesh] OR "Gender Based Violence"[Mesh] OR “intimate partner violence”[Mesh] OR “domestic violence”[Mesh] OR “rape”[Mesh] OR “empowerment”[tiab] OR “agency”[tiab] OR “autonomy”[tiab]

AND

“Health System Plans”[MESH] OR “health information systems”[MESH] OR “Delivery of healthcare”[MESH] OR “Health Workforce”[MESH] OR “Health Financing”[MESH] OR “health system*”[tiab]  OR “health care system*”[tiab] OR “health care”[tiab] OR “health care sector*”[tiab] OR “healthcare delivery system”[tiab] OR “primary health care”[tiab] OR “health service*”[tiab] OR “health care service*”[tiab] OR “delivery of health care”[tiab] OR “healthcare delivery”[tiab] OR “service delivery OR health workforce”[tiab] OR “health workers”[tiab] OR “human resources for health”[tiab] OR “HRH”[tiab] OR “health information systems”[tiab] OR “health management information systems”[tiab] OR “HIS”[tiab] OR “HMIS”[tiab] OR “health system governance”[tiab] OR “health system leadership”[tiab] OR “health financing”[tiab] OR “health system financing”[tiab] OR “medical products”[tiab] OR “essential medicine*”[tiab] OR “health supply chain”[tiab] OR “Health system supply chain”[tiab]
