# Supplementary material for: Towards a common understanding of gender-responsive monitoring and evaluation for health programs and interventions: Evidence from a scoping review
Source: SSM Health Syst. Author manuscript; Available in PMC 2026 Feb 27. (PMC12945375; doi:10.1016/j.ssmhs.2025.100059)
Supplement: Supplemental Material File 2 [file NIHMS2149056-supplement-Supplemental_Material_File_2.docx]

Supplemental Table 1: Key findings of included articles

| Authors | Countries of focus | RMANCAH issue(s) examined | Research design | Research Scope | Ways in which gender is integrated into M&E |
| --- | --- | --- | --- | --- | --- |
| Abramsky 2016 | Uganda | Violence against women, sexual health | Evaluation of an intervention to mobilize community members to prevent violence against women  Cluster randomized control trial run between 2007 and 2012  64 sites at baseline and 96 at endline were chosen to be sampled from by intervention-trained “community activists.” Community activists then randomly sampled 35 households per site | 1,583 participants at baseline and 2,532 at endline | *Community participation*  Trained “community activists” engaged in study design  *Use of established tools*  Gender-based indicators based on instruments used in the WHO Multi-country Study on Women’s Health and Domestic Violence and the Uganda Demographic and Health Survey  *Use of relevant theory*  Included sender theoretical models: socio-ecological model; ecological model of violence; focus on community and individual levels  *Sex disaggregated data*  Analysis was performed separately between men and women. The associations between each intermediate outcome and women’s past year experience of physical IPV and men’s past year perpetration of IPV was then explored  *Gender responsive indicators*  “Women’s past year experience of physical IPV”  “Men’s past year perpetration of IPV”  “Okay for others in community to intervene if they know IPV is occurring,  “People who have witnessed/heard violence who have responded appropriately”  “Acceptable for a man to use violence against his partner”  *Other gender considerations*  Because earlier studies suggested males may underreport IPV, an anonymous pen and paper survey respondents would fill out at the end of the interview  Focus on experience of physical and sexual IPV, male and female attitudes towards the acceptability of IPV, reductions in past year sexual concurrency among men |
| Adamou 2019 | N/A | Family planning | Desk review of published peer-reviewed and gray literature on male engagement in FP  Key informant interviews conducted with experts identified as relevant in the desk review  Set up an “online forum” where experts in male engagement in family planning were able to share their experiences with indicators and validate key themes revealed in the desk review and KIIs | 293 publications found, 72 included for review  14 key informant interviews conducted  42 experts joined the online forum | *Gender responsive indicators*  15 indicators for measuring and evaluating male engagement in family planning identified and grouped into three theoretical categories  Men as clients (e.g.)   - “Men’s condom use at last sex” (outcome) - “Number/percent of vasectomy referrals” (output) - “Number of FP providers trained on male-specific FP” (output)   Men as partners (e.g.)   - “Percent of men who share in the decision making of RH issues with their spouse or sexual partner” (outcome) - “Percent of men who disagree that contraception is a woman’s business and a man should not have to worry about it” (outcome) - “Evidence of engagement of men in FP incorporated in national health standards or policies” (outcome)   Men as agents of change   - “Attitudes towards gender norms (utilizing the GEM Scale)” (impact) - “Number of providers trained on gender equity and sensitivity” (output) - “Number of national level programs/ policies/advocacy campaigns that address gender equity” (outcome)   *Use of gender scores*  The Gender Equitable Men (GEM) Scale is an essential tool that has not been integrated into male engagement family planning monitoring and evaluation. This scale can be used well in conjunction with qualitative methods to gain more insight into knowledge, attitudes and practices  *Other gender considerations*  Qualitative data are often required to complement quantitative measurements because few indicators for male engagement in FP are collected through routine data collection. However, qualitative tools are also essential to understanding the success of programs and male behavior.  Relying on Demographic Health Survey to assess FP outcomes is challenging because questions on whether men have accessed FP services are not included in the main questionnaire. |
| Agam-Britton 2018 | Israel | Adolescent health | Evaluation of an intervention to improve universal wellness practices for adolescents  RCT evaluated a girls-only group and a mixed-gender group given a wellness intervention | 259 students were randomly assigned to the control group (no intervention given), girls only, and mixed-gender groups  75 students from each group were assessed at endline | *Sex disaggregated data*  Utilized sex disaggregated data by evaluating the effectiveness of girls only vs mixed gender groups  *Gender responsive indicators*  Included girl-focused self-esteem and body image outcomes including Self-esteem, media literacy, Sociocultural Attitudes Towards Appearance, Drive for thinness and body dissatisfaction, Body-esteem |
| Bartels 2019 | Lebanon | Sexual health | Evaluation of programs seeking to aid refugee women and girls experiencing gender based violence  Survey using the Sensemaker tool was given to refugee women and girls identified by aid programs utilizing their gender-based violence programs  The survey was created through a two-day workshop where 30 members of evaluated organization drafted questions and “dyads” that SenseMaker utilizes to analyze data | Pretested with 40 women, 198 participated across five refugee locations between May and August 2018 | *Use of established tools*  Cognitive Edge’s SenseMaker program was the core tool used. SenseMaker is an online program that collects micronaratives from participants through filling out an online app. SenseMaker is a mixed methods tool as it elicits qualitative narratives quantitative from participants and insights through correlating data between participants. Utilizing audio recordings to respond to prompt and easy to use survey, SenseMaker is able to gain a nuanced perspective of the participants experience with gender based violence  SenseMaker was used to explore benefits from the GBV program, safety, feelings after program utilization, empowerment and decision making through programs, motivation for accessing programs, effectiveness of group activities and financial assistance, and feelings while participating in activities |
| Berti 2015 | Honduras | Maternal Health | Evaluation of the intervention REDES intervention aiming to improve male engagement and empowerment of women surrounding maternal health  Cluster randomized control trial administered through a household survey | At baseline and endline, 30 cluster samples of communities selected from 229 participating communities, 10 households with at least one mother-child pair in each cluster were selected to take survey  299 responses total | *Use of established tools*  Survey tool modeled on findings of the Child Survival Technical Support Project at Johns Hopkins University, adapted to the project context  101 survey questions covered household demographics, prenatal and postnatal maternal care birth and delivery care, mothers’ perception of the father’s role in pregnancy, family planning, and child health  *Gender responsive indicators*  Utilized gender-responsive indicators (e,g.)   - - “Mother knew pregnancy danger signs”   - “Mother took micronutrient supplements”   - “Parents developed a delivery plan”   - “Institutional delivery”   - “Mother attended postnatal checkup”   *Use of gender scoring*  Utilized gender scoring to analyze variables such as “Father’s score”(A, B, or C). Father’s were scored on responses to questions regarding interest in accompanying mothers to checkups and birth and their interest in the content of the checkup. Based on the response, fathers could earn an A, B, or C, score with A being optimal engagement. |
| Blanchard 2013 | India | Sexual Health | Evaluation of an intervention aiming to prevent HIV amongst female sex workers (FSWs) through community mobilization and empowerment  Secondary analysis of a representative behavioral tracking survey conducted among FSWs  Conventional cluster sampling was used in locations where FSW population was consistent over time, while time-location cluster sampling was used in locations where FSWs solicited work  Data was collected by researchers using interview-based culturally sensitive and contextual questionnaires | 1,750 FSWs participated in the study | *Use of relevant theory*  An “integrated empowerment framework” was the basis for the empirical examination of the path to empowerment as an outcome and as a means to create social transformation. Three “empowerment domains” were created:   - - “Power within” (self-esteem and confidence)   - “Power with” (collective identity and solidarity)   - “Power over” (access to social entitlements)   *Gender responsive indicators*  Created “empowerment variables” that weighed composite indicators for each domains through Principal Component Analysis. These composite variables were based on a +/- scale ranging between +2 to -2 that correlated with “strongly agree” to “strongly disagree” responses on the survey  “Proximate measures of social and personal transformation” were created using Principal Components analysis and five components were created:   - - “autonomy and violence or abuse from more powerful groups for social transformation”   - “self-efficacy for condom use with clients”   - “self-efficacy for condom use with regular partners”   - “self-efficacy for service utilization to reflect personal transformation” |
| Bliznashka 2022 | Burkina Faso | Women’s Health, Child Health and Nutrition | Evaluation of an intervention to improve women and child health through empowerment in a gender sensitive agricultural program  Cluster randomized control trial administered at baseline and endline between 2010 and 2012  55 villages were split into a control group and two groups where the intervention was implemented by women health leaders or a community health committee | 1767 households and 1882 women sampled | *Gender responsive indicators*  Gendered indicators focusing on:   - - Women’s underweight   - Empowerment   - Infant and young child feeding   - Hygiene knowledge |
| Burke 2019 | Mozambique | Adolescent Health, Sexual Health | Evaluation of a social and economic empowerment intervention to reduce HIV vulnerability amongst girls  In depth interviews and focus groups conducted in two rounds one year apart  Participants randomly sampled from 12 communities randomly sampled from all communities that received the intervention | 96 girls, 48, household heads, and 72 influential males interviewed between the two rounds  24 groups of community members participated in focus group discussions between the two rounds | *Use of established tools*  Semi-structured interview guides and FGD guides for girls, heads of household, influential males, and community covered topics such as (e.g.)   - - “Perception of the intervention”   - “Girls’ experience earning money and what they spent it on”   - “Whether respondents thought girls’ relationships had changed, including with household and family members, community members, friends, and sexual partners”   *Use of relevant theory*  Used the social ecological model (SEM) to design the qualitative evaluation and analyze the data |
| Colombini 2021 | South Africa, Tanzania | Sexual Health, Adolescent Health | Evaluation of a program aiming to implement GBV screening into HIV counseling and testing amongst adolescent and young women celled EMPOWER  Field notes from 10 randomly selected sessions included as participant observation  In-depth Interviews conducted with EMPOWER participants 3 months after enrollment  Semi-structured interviews conducted with clinical staffers | 39 EMPOWER participants (25 South Africa; 14 Tanzania)  13 clinicians (10 South Africa; 3 Tanzania) | *Gender responsive monitoring integration into health services*  Collected process data on the number of women screened, screening outcomes, and the proportion referred to further services  Clinical staff was trained on a variety of monitoring and screening of GBV skills including (e.g.)   - - “GBV identification and risk assessment, first response and referral procedures”   - “GBV screening and risk assessment job aid was developed including 6 questions for identification and 5 for risk assessment”   - “Warm referral (study staff directly contacting support services for appointment)” |
| Dev 2019 | Kenya | Family Planning | Evaluating of a mobile health family planning tool for postpartum women  Cross sectional qualitative study conducted at four sites (two urban and two rural)  Postpartum women recruited in waiting areas of infant immunization clinics  In-depth interviews with postpartum women and semi-structured interviews with FP workers conducted by authors | 25 postpartum women and 17 FP providers were interviewed | *Use of relevant theory*  Intervention and evaluation were guided by the Theory of Planned Behavior, the Health Belief Model, and the Social Cognitive Theory |
| Exner-Cortens 2021 | Canada | Sexual Health, Adolescent Health | Evaluation of an intervention aiming to improve young male empowerment, structural awareness, and sexual behaviors  Teens recruited in a high school through personal signup  Participants took photos then met in groups to discuss them.  Follow-up IDIs with participants were conducted by graduate assistants | 6 adolescent boys with an average age of 14 participated in the intervention | *Community-based participatory methods*  The photo-based evaluation method of the intervention is designed to produce participant-led data collection and scholar-led data analysis  The photos taken during the evaluation were discussed in group conversations to elicit what they revealed about the intervention and how participants see the world different after  *Use of relevant theory*  The premise of the evaluation and the development of the tools was based on Drew’s 2010 outline of feminist evaluation practice in the gendered experiences of men and boys  The presentation of the photos to the group and creation of knowledge about what the intervention achieved through this practice was adapted from the SHOWeD method developed by (Shaffer 1983), which is based on Paolo Freire’s theories of change through pedagogical praxis (1973)  *Use of relevant tools*  Based on these theoretical tools, the pictures and following conversation attempted to elicit:   - - Why the participants chose the photos they did to share   - What is happening in the photos that is reflective of their learning   - How the photos related to the intervention |
| Figueroa 2016 | Mozambique | Sexual Health | Evaluation of an intervention attempting to change gender and sexual norms surrounding HIV prevention using community dialogue  Participants systematically random sampled from those who participated in the intervention in a selected region  Control sample was drawn from a list of people who agreed to participate in the intervention but had not yet  Survey given by interview questionnaire | 915 participants in the post intervention survey, about half who received the intervention and half who did not | *Gender responsive indicators*  Questionnaire included questions eliciting about HIV (e.g.)   - - “HIV partner communication”   - “HIV prevention knowledge”   - “HIV stigma”   *Use of gender scores*  Gender attitudes were measured through a series of 12 statements on gender roles. This ​​was analyzed through a Likert-type scale of total disagreement to total agreement between genders.  A gender equity attitude scale was developed to assess the number of tasks reported done by both men and women |
| Ghanotakis 2016 | Uganda | Sexual Health, Family Planning | Evaluation of an intervention aimed at improving family planning and HIV prevention through altering male engagement and gender norms  Cross sectional survey implemented at baseline and six months later  Qualifying men must have completed the baseline survey and one class with male role models as part of the intervention | 1,251 were surveyed at baseline and 1,122 were surveyed at endline | *Community-based participatory methods*  32 male role models assisted in the administering of the surveys at both baseline and endline  Results were interpreted with district health officials, health facility managers, local government chiefs, and all 32 model men in meetings at the district offices  *Use of gender scores*  The Gender Equitable Men (GEM) Scale was used to structure the survey and assess men’s understandings of gender norms.  Used 23 of the GEM Scale’s 24 items examining 5 areas indicating gender norm understanding:   - - “violence”   - “Sexual relationships”   - “Reproductive health and disease prevention”   - “Domestic chores and childcare”   - “Relationships with other men”   5 point Likert scale from strongly agree to strongly disagree based on related prompts then were aggregated to produce total score gender norms understanding |
| Khoza 2018 | South Africa | Sexual Health, Adolescent Health | Evaluation of an intervention using cash transfers to address sexual health issues amongst adolescents  Participants interviewed 6 months and one year after intervention start | 49 participants (30 male, 19 female) in the intervention were interviewed about their experiences with the cash transfers | *Gender disaggregated data*  Interviews were intentionally analyzed to compare the experiences between male and female participants in the study |
| Kowalcyzk 2015 | N/A | N/A | Systematic literature review of issues related to the planning, implementation, and evaluation of gender-based programs | 5467 abstracts screened  565 relevant  123 met eligibility criteria and included in the extraction | *Use of relevant theory*  One-quarter of the studies specifically utilized a conceptual model or theory that guided the research. Of these the Social Cognitive Theory, Grounded Theory, and Cognitive Behavioral Theory were the most common  Other studies used various models, including gender-specific models, family-specific models, and HIV/AIDS-specific models.  *Gender responsive indicators*  Indicators extracted from eligible articles include   - - “improved perceptions of gender norms within a community”   - “increased knowledge within a community of gender inequities”   - “increased involvement of local decision-makers and other community leaders in women's health issues”   - “Increased community capacity to address women and girls’ health” |
| Krishnan 2016 | India | Sexual Health | Evaluation of an intervention aimed at improving workplace gender equity  Anonymous cross sectional surveys given to randomly sampled employees two factories (one control and one experimental) at baseline and endline (one year later)  Surveys were administered by an interactive voice response system | 926 (428 baseline and 498 at endline) were surveyed | *Use of relevant theory*  Intervention evaluation was guided by the Social Cognitive Theory  *Gender responsive indicators*E.g.   - - “gender equitable attitudes”   - “acceptability of IPV towards a spouse”   - “attitudes towards alcohol”   *Use of gender scoring*  Perceptions of gender equity were assessed by an abbreviated version of the Gender Equity Scale for Women (GESW) with a high score being more favorable gender attitudes |
| Lecroy 2018 | USA | Adolescent Health, Sexual Health | Evaluation of an intervention attempting to reduce sexual risk factors in adolescent girls  Girls randomly sampled from middle schools and community centers in urban Southwest USA locality then randomly placed into control and experiment groups  Survey administered at baseline, 6 months, and 18 month endline | 801 adolescent girls sampled | *Gender responsive indicators* E.g.   - - “condom technical skills”   - “condom self-efficacy”   - “STD knowledge”   *Use of gender scoring*  The “girl efficacy” measure was developed to assess the intervention’s ability to increase confidence. The measure included four Likert items such as “I am a confident girl.” rated from “strongly disagree” to “strongly agree.” |
| Lees 2021 | Tanzania | Women’s Health | Evaluation of an intervention addressing intimate partner violence through gender transformative approaches  Qualitative research conducted through hearsay ethnographies and then in-depth interviewing and and focus group discussions at the end of the intervention | 10 community members (5 men, 5 women) conducted hearsay ethnographies over 5 years  20 IDIs conducted post intervention (6 women, 14 men)  16 groups with a total of 160 participants (40 of each: men, women, girls, and boys) | *Community-based participatory methods*  Hearsay ethnographies–daily diaries about relevant IPV activities in the locality–were the primary data collection tool, meaning that community members were largely responsible for evaluation data  Community ethnographers were visited by trained team members bimonthly to get new notebooks  *Use of relevant theory*  Theoretically grounded in and directly mentions the work of Judith Butler  *Gender responsive indicators*  Qualitative analysis approaches used a “gender lens” which focused on the following aspects of IPV:   - - “gendered explanations of violence"   - "awareness of masculinity as a system of power and violence"   - "undermining women's value to decision-making' |
| Long 2022 | Mexico | Adolescent Health | Evaluation of an intervention aiming to increase menstrual health literacy among adolescent girls  Cross-sectional quantitative study that randomly sampled teenagers at middle schools | 140 girls surveyed at baseline and 193 at endline | *Use of established tools*  Adapted the menstruation related engagement and self-efficacy and reduced menstruation related stress (MENSES) tool to measure intervention impact  MENSES items measure frequency (almost always, sometimes, and never), strength (a lot, a little, and never), and agreement (strongly agree, agree, disagree, and strongly disagree)  45 questions asked across three domains:   - - Engagement–school participation experiences during menstruation   - Stress–menstrual related fears or worries a girl may have experienced the last time she managed her menstruation while at school   - Self efficacy–measures girls’ beliefs in their own ability to do a menstrual-related task or school activity when menstruating at school |
| Mandal 2017 | N/A | Women’s Health | Systematic review of family planning and maternal health program evaluation to elicit measures of women’s empowerment and other constructs | 2,450 articles found, 1,741 included for review  196 included for full review  16 articles extracted from | *Use of established tools*  Gender Equitable Men (GEM) scale and the Sexual Relationship Power Scale named as key scales to measure gender based violence and women’s empowerment program evaluations  *Gender responsive indicators*  No study included in the review had a complete set of indicators in terms of level of operation and women’s empowerment measures. Most studies did include:   - - “autonomy and empowerment as a process”   - “measures of women’s autonomy (e.g., decision-making power, economic capabilities and assets)”   - “measures of determinants of women’s autonomy (e.g., confidence/self-esteem)”   - Measures of changes over time   Other indicators found included (e.g.):   - - “Gender-equitable attitudes regarding sexual and reproductive health”   - “Gender-equitable attitudes regarding domestic matters”   - “Support in pregnancy and health-seeking behaviors”   - “Attitudes around women’s health”   - “Provider-client interaction”   *Use of gender scores*  A number of gender scales were identified as being useful to measure reproductive empowerment in more developed countries, including (e.g.)   - - Reproductive Autonomy and Reproductive Coercion scale   - Relational Response to Condom Use scale   - Supportive Behavior Questionnaire scale   - Salutation, Assessment, Help, andReassurance index   - Autonomy index |
| Muraya 2017 | Kenya | Maternal and Child Health and Nutrition | Evaluation of gendered decision making in child nutrition interventions  Qualitative study through a descriptive questionnaire given by two community-recruited data collectors  15 households purposively sampled from a health center with a highly active nutrition program | 95 total participants | *Community-based participatory methods*  Two field staff from the community were primary data collectors and administered the qualitative survey  These data collectors were also included in data analysis  Preliminary data was shared with participants for feedback in household visits which aided researcher interpretation of the data |
| Pulerwitz 2010 | N/A | Sexual Health | Evaluation report for an intervention focused on male engagement in HIV implemented on three different continents  Programs were evaluated using both qualitative and quantitative methods | 10 interventions conducted in South Asia, Latin America, and Sub-Saharan Africa | *Creation of relevant tools*  Created the Gender Equitable Men (GEM) scale to measure intervention improvement of men’s viewpoints and norms surrounding 24 items, including:   - - “women’s and men’s roles in domestic work and child care”   - “sexuality and sexual relationships”   - “reproductive health and disease prevention”   - “violence”   - “homophobia and relations between men”   *Gender responsive indicators*  Additional indicators were used across sites such as (e.g.)   - - “Communication between partners about HIV”   - “Condom use” |
| Sahyoun 2019 | Lebanon | Maternal and Child Health and Nutrition | Evaluation of an intervention aiming to improve nutrition through community kitchen social enterprises  Quantitative evaluation was done by questionnaires given at baseline and endline  Qualitative evaluation was done through semi-structured interviews at the end of the study  Recruitment was done by social workers working inside the camp | 32 women were sampled | *Use of established tools*  The Women’s Empowerment in Agriculture Index (WEAI) was used to create and analyze the quantitative data to asses:   - - “access to and decision-making power over income”   - “decisions related to meal planning”   - “visits to family or relatives"   The Mental Health Inventory (MHI-5) was used to assess mental health on a scored scale.  The Duke Social Support Index (DSSI) and its 10 items were used as an assessment for participant’s level of social support  *Gender responsive indicators* E.g.   - - Decision making and social support   - Gender economic independence   - Skill acquisition   - Personal growth |
| Sanytha 2019 | India | Sexual Health | Evaluation of an intervention aiming to transform young male attitudes about gender roles and violence  Cluster randomized control trial focusing on boys enrolled in 30 sports clubs, then evenly divided into a control and experiment group | 1,149 young men surveyed at baseline  1,033 young men surveyed at endline | *Use of gender scoring*  Used the Gender Equitable Men (GEM) Scale to create questions on gender roles. Gender role and attitudes and notions of masculinity were scored from 0(least equitable)-9 (most equitable)  *Use of established tools*  Survey questions and other composite scoring of responses were shaped by the WHO multicountry study on violence and the National Family Health Survey  *Gender responsive indicators* E.g.   - - "men in their community justify wife beating in at least one situation”   - “percentage reporting that their friends condone violence against women and girls in at least one situation”   - “percentage who watch pornography" |
| Schuster 2019 | Palestine | Sexual Health | Monitoring community norms surrounding women’s empowerment  Cross sectional survey given by members of local organizations  Participants sampled in public areas | 392 total people responded to the survey | *Community based participatory methods*  Trained members of local civil society organizations to administer interview  *Use of relevant theory*  Cultural Consensus Theory–which postulates that culture is shared knowledge stored collectively in the individual minds of societal participants–was the basis of the monitoring tool  *Use of established tools*  Cultural Consensus Analysis (CCA) was the central tool to assess norms. Focusing on assessing gender empowerment on the community’s own terms. The CCA tool utilized here was created by a team led by anthropologists  Tool components were created from a literature review, sources focused on GBV and women’s economic empowerment norms, EPJP program documents, and the USAID/West Bank and Gaza Gender Analysis  *Gender responsive indicators*  Topics of statements participants responded to include (e.g.):   - - GBV   - Economic empowerment of women   - Household and community dynamics of GBV   *Sex disaggregated data*  Part of the analysis disaggregated statement responses by men and women and compared results |
| Seff 2021 | Democratic Republic of the Congo | Maternal and Adolescent Health | Evaluation of an intervention attempting to change caregiver attitudes to improve the safety of adolescent girls  Secondary analysis of randomized control trial data surveying caregivers and young adolescent girls  Questionnaire given through computer assisted interview softwares at baseline and endline one year apart | 732 girls and caregivers surveyed | *Use of relevant theory*  The Social Ecological Model was used as a framework to understand the results of the trial particularly concerning intersectionality between caregiving, the refugee setting, and poor school quality  *Sex disaggregated data*  Data was disaggregated by sex for caregiver-specific responses and analyzed for their differences  *Gender responsive indicators*  Predictors of interest included (e.g.)   - - “caregivers’ gender-equitable attitudes”   - “gender roles and dynamics”   *Use of gender scores*  Caregivers were assessed through a gender-equitable attitude score based responses to 10 agree-disagree statements. Scored from 0-10, 10 being the most equitable attitudes  *Other gender considerations*  Separate surveys were given for caregivers and girls  Sensitive questions around marriage and sexual violence were not given to participants between 10-12 years old  All data was collected in private rooms provided by a local-community based organization  More sensitive questions for girls such as those on sexual behaviors or violence were collected through auto-computer assisted self-interview software |
| Sharma 2020 | India | Maternal and Child Health | Evaluation of an economic empowerment intervention for women in India  Tracked indicators and outcomes of enrolled young women through an online management system  Endline assessment was conducted with in-depth interviews and focus group discussions with purposively recruited participants | 37,324 women tracked through online system  12 FGD conducted with participants, peer educators, mothers-in-law, outreach workers, and community health workers  22 IDIs conducted with husbands, midwives, medical officers, district bureaucrats, community health workers, and peer educators | *Community based participatory methods*  Community outreach workers collected routine data  *Gender responsive indicators* E.g.   - - “awareness on maternal and child health care”   - “awareness on national health insurance and MGNREGA schemes”   - “utilization of maternal and child health services” |
| Sharma 2022 | N/A | Sexual Health | Systematic mapping and in-depth desk review of gray literature and published articles on the monitoring and evaluation of GBV programs in humanitarian settings  IDIs and focus group discussions conducted with key professionals in the area  Participants sampled through purposive and snowball methods | 2,108 documents identified  311 included in review  145 articles extracted from  11 semi-structured IDIs conducted  5 FGDs conducted | *Community based participatory methods*  Important facets of community based and participatory methods elicited from the interviews and reviewed articles include:   - - Building partnerships with key actors and stakeholders, including local groups and organizations   - Consideration of diversity factors within the affected populations. M&E should be developed with relevant subgroups such as adolescent girls, the elderly, people living with disabilities, and LGBTQI folks as well as with recognition of intersectionality that compounds GBV   - Women and girls in the locality must actively be involved in the design of monitoring and evaluation activities   - Participatory methods are important tool to ensure these women and girls are included in design   - Mechanisms for the community to give feedback must be established   - Community should always consent to M&E activities, especially when activities may differ from community norms and expectations"   - M&E tools should be field-tested with the community   - Inclusive participation requires carrying out data collection at different times of the day ad potentially at the homes of participants   - Critical to understand, respect, and use local understanding of GBV-related concepts. Community must be worked with to ensure the right terms and contextual indicators are used in a culturally sensitive way   *Data collector ethics*  Interviews and reviewed articles stressed that:   - - Staff collecting data and involved in M&E should be trained on psychological first aid   - M&E staff should be prepared with resources for self-care tactics as they may experience secondary trauma   - Gender parity should be ensured among staff   - Same-sex interviews are a key practice, particularly for young girls and older male participants   - Non-GBV specialists should not ask direct questions about experiences of GBV   *Sex disaggregated data*  Disaggregation of data by sex was a key tool identified in the interviews and desk review  *Gender responsive indicators*  Interview and desk review data asserted that GBV risk indicators should be developed through participatory processes with input from local staff and women and girls themselves but in alignment with established tools.  Proxy indicators should be leveraged to better understand GBV risk while not directly asking about GBV personal experience  *Other gender considerations*  Data collection should not include asking direct questions regarding community or individuals’ personal experiences of GBV |
| Shimpuku 2019 | Tanzania | Maternal and Child Health | Evaluation of an intervention attempting to improve birth preparedness through family-oriented education  Cross sectional evaluation with an intervention and control groups | 128 total participants (64 in each group) | *Gender responsive indicators* E.g.   - - “Desired place of birth”   - “preferred birth attendant”   - “location of the closest facility for birth and in case of complications”   - “An identified labour and birth companion”   - “An identified support to look after the home and other children while the woman was away” |
| Spiezer 2018 | South Africa | Sexual Health | Evaluation of an intervention aiming to improve sex risk behaviors  Men who drink alcohol recruited outside places where alcohol is served  Recruited men and their sexual partners completed surveys based on their placement in one of two trial groups and one control group | 267 couples surveyed and tracked from baseline to endline | *Sex disaggregated data*  Sex disaggregation was an important tool utilized as the unit of focus was couples. Surveys were analyzed as men only, women only, and occasionally both then compared across sex  *Gender responsive indicators*   - E.g.   - “consistent condom use”   - “partner communication”   - “gender norms” |
| Tura 2020 | Mozambique | Maternal Health | Evaluation of an intervention attempting to increase women’s agency and maternal health service usage through community-based savings groups  Two-stage cluster sampling process breaking women into an intervention group and a control group | 205 total participants | *Community based participatory research*  Evaluation was designed in conjunction with and largely conducted by a local NGO  *Use of established tools*  Survey was created from the Mozambique Demographic and Health Survey and General Self-Efficacy Scale  *Use of gender scores*  Five-item scale was used to assess women’s generalized self-efficacy. Agreement was assessed through a Likert-type scale from 1 (“strong disagreement”) to 5 (“strong agreement”)  *Gender responsive indicators*  Agency was assessed through decision-making autonomy and self-efficacy, which included a variety of indicators (e.g.):   - - “who usually makes decisions about the respondent’s health care”   - “who usually makes decisions about family planning”   - “Who usually makes decisions about large household purchases” |
